# Gene–Environment Interplay Between Residential Walkability and BMI: A Finnish Twin Study

**DOI:** 10.1002/oby.70191

**Published:** 2026-04-27

**Authors:** Zhiyang Wang, Bram Berntzen, Nishit Patel, Stephanie Zellers, Sari Aaltonen, Danielle Dick, Karri Silventoinen, Jeroen Lakerveld, Jaakko Kaprio

**Affiliations:** ^1^ Institute for Molecular Medicine Finland, Helsinki Institute of Life Science, University of Helsinki Helsinki Finland; ^2^ Department of Epidemiology and Data Science, Amsterdam UMC Amsterdam the Netherlands; ^3^ Amsterdam Public Health Research Institute Amsterdam the Netherlands; ^4^ Upstream Team, Amsterdam UMC Amsterdam the Netherlands; ^5^ Department of Psychiatry Rutgers University Piscataway New Jersey USA; ^6^ Helsinki Institute for Demography and Population Health, University of Helsinki Helsinki Finland

**Keywords:** BMI, gene, twin, walkability

## Abstract

**Objective:**

BMI is influenced by both genetic and environmental factors, including features of the obesogenic environment such as walkability. This study investigated the interplay between residential walkability, genetics, and BMI among 4312 individuals from two Finnish twin cohorts (mean age: 43).

**Methods:**

Residential walkability was quantified using a composite index including built and natural environmental features.

**Results:**

Multiple linear regression, adjusted for sociodemographic factors, showed that higher walkability was associated with lower BMI (coefficient: −0.02, 95% CI: −0.04, −0.01), but pairwise twin analyses indicated that this association was not independent of genetic and early shared environmental influences. Univariate twin modeling revealed that additive genetic effects accounted for 22% of the variation in walkability when participants were middle‐aged. Bivariate moderation twin modeling showed that the additive genetic influence on BMI was significantly stronger among individuals living in areas with average or moderately low residential walkability. The unique environmental component played a larger role among those living in areas with either very low or very high residential walkability.

**Conclusions:**

These findings underscored the complex gene–environment interplay between walkability and BMI. While genetic susceptibility cannot be modified, public health strategies that focus on the obesogenic environment, such as improving walkability, may still help mitigate obesity risk.

## Study Importance

- What is already known?
  ○ Low residential walkability is linked to higher risks of overweight and obesity in adults, but these associations may be influenced by genetic factors.
- What does this study add?
  ○ In Finland, higher walkability was associated with lower BMI but not independently of genetic and early shared environmental influences.
  ○ Genetic effects on BMI were stronger among middle‐aged adults living in areas with average walkability, while environmental influences dominated in areas with very low or very high walkability.
- How might these results change the direction of research or the focus of clinical practice?
  ○ Considering genetic susceptibility is recommended in studies of the obesogenic environment.
  ○ Improving walkability may still help reduce obesity risk, even among genetically predisposed individuals.

## 1 Introduction

Body mass index (BMI) over 30 is the most widely used indicator of obesity, and it is associated with the risk of over 200 diseases [1]. Recent data from the Finnish Institute for Health and Welfare (THL) show that nearly one in four adults in Finland is currently living with obesity [2], and this prevalence is projected to rise to 31% by 2028 [3]. Globally, the number of adults living with overweight or obesity is projected to reach 3.8 billion by 2050, accounting for more than half of the world's expected population [4]. In 2014, obesity alone accounted for up to 8% of healthcare expenditures in EU member states, and the socioeconomic burden will be heavier if considering the risk of being overweight also [5]. According to the World Obesity Atlas 2023, the global economic impact of overweight and obesity is expected to reach $4.32 trillion annually by 2035 [6].

Both genetic and environmental factors play a significant role in the development of obesity. A previously conducted large‐scale twin meta‐analysis showed that 30%–40% of the variance in BMI was attributed to environmental influences among middle‐aged and older adults, whereas the rest of the variation was explained by genetic differences [7]. The concept of the obesogenic environment has emerged to describe how modern living conditions contribute to the global obesity epidemic [8]. Residential walkability, defined as the extent to which the living environment is conducive for walking, is a vital aspect of the obesogenic environment [9]. A 2019 systematic review found that less walkable neighborhoods are associated with overweight and obesity among adults in North America and Australia (in the majority of included studies) [10], but similar evidence from Europe, in particular Nordic countries such as Finland, remains limited. European cities usually have a better level of walkability than cities in the United States and Australia [11]. Improving walkability has become widely adopted as one of the instruments in creating an inclusive, safe, and sustainable living environment [12], aligned with the UN Sustainable Development Goal of good health and well‐being by 2030.

At the same time, the importance of genetics in BMI should not be neglected, which makes the mechanism of the effect of walkability on BMI more complex. First, there is a genetic contribution to the living environment people choose. Based on the middle‐aged adults from the Washington State Twin Registry (WSTR), the heritability of residential‐level walkability was estimated to be 19%, and walkability protected against high BMI via the genetic pathway [13]. Certain factors, like education level and income, that affect choosing the residential location are also affected by genetics [14, 15]. Moreover, how individuals experience and engage with their surroundings, such as the frequency of visiting green spaces, was shown to be partially heritable [16]. Second, the effect of the obesogenic environment on the risk of suboptimal BMI varies by genotype or vice versa, in a gene–environment interaction (G × E). The WSTR study indeed found that walkability suppressed the genetic risk for high BMI [13]. However, findings from Dutch adolescents showed no significant interaction between genetic risk and walkability on the change in BMI [17]. These results warrant more studies to elucidate the interplay between walkability, genetics, and BMI. Twin design studies are especially suitable, as they employ a natural quasi‐experimental framework to disentangle genetic and environmental influences and their interplay by comparing phenotypes' similarities between monozygotic (MZ) and dizygotic (DZ) twins. Furthermore, shared similar environmental factors during early life (e.g., in utero, family/cultural, neighborhood environment) are controlled for to enhance causal inference, because twins have been mostly reared together.

This study aims to elucidate the interplay between residential walkability, genetics, and BMI in Finnish middle‐aged adult twins, with the following three objectives: (1) to examine the association between residential walkability and BMI and whether the association is independent of shared genetic and early‐life environmental factors, (2) to estimate the extent to which the genetic component contributes to population variation in residential walkability, and (3) to assess whether the residential walkability moderates the relative genetic and environmental contributions to BMI.

## 2 Methods

### 2.1 Participants

This study draws the data from the latest cross‐sectional follow‐ups of two Finnish twin cohorts: FinnTwin16 and FinnTwin12. FinnTwin16 is a nationwide prospective cohort of all Finnish twins born between 1975 and 1979. The latest follow‐up in 2024 was conducted at their mid‐40s (mean age: 47.2) with retention rates of 44%. A total of 2530 participants were included. The prospective FinnTwin12 cohort includes all Finnish twins born between 1983 and 1987. Its latest follow‐up in 2022 surveyed participants at their early midlife (mean age: 37.2) with a 41% retention rate and 2122 participants. Zygosity was determined using DNA polymorphism analysis and/or a validated questionnaire [18]. Recent publications have described the updates of the two cohorts [19, 20].

The ethics committee of the Department of Public Health of the University of Helsinki (Helsinki, Finland) and the Institutional Review Board of Indiana University (Bloomington, Indiana, USA) approved the FinnTwin16 and FinnTwin12 study protocols from the start of the cohort. The ethical approval of the HUS Regional Committee on Medical Research Ethics covers the most recent data collections (FinnTwin16; HUS/1584/2024, dated April 22, 2024, and FinnTwin12; HUS/2226/2021, dated September 22, 2021). All participants and their parents/legal guardians gave informed written consent to participate in the study.

### 2.2 Measures

BMI (weight in kilograms divided by height in meters squared) was calculated using self‐reported height and weight from the latest follow‐up in two cohorts (mid‐40s in FinnTwin16 and early midlife in FinnTwin12). If height was missing, values from earlier follow‐ups were used, as the adult height is quite stable. Participants without BMI information were removed from the analysis (*n* = 27 in FinnTwin16 and *n* = 9 in FinnTwin12).

Residential walkability was assessed using a composite index based on seven core components: walkable street length, intersection density, green spaces, slope, public transport access, land use mix, and population, derived from harmonized, high‐resolution datasets, including Sentinel‐2, NASA's elevation models, OpenStreetMap, CORINE Land Cover, and other secondary geospatial sources. A 100 m × 100 m hierarchical grid system and advanced geospatial techniques were applied at scale to model real‐world density and proximity effects. The index was weighted by population. A higher level of residential walkability indicates stronger street connectivity and diverse land use. A detailed description of the development and content of the walkability index is provided elsewhere [21]. The residential histories of two cohorts from birth were derived from the Digital and Population Data Services Agency, Finland, and were used to merge the residential walkability. The geocodes of the residential addresses in 2018 were initially used to merge. If the 2018 address was unavailable, we substituted it with the 2017 address, and if still unavailable, with the 2019 address. Therefore, there is no large temporal gap between the assessment of residential walkability and BMI. Participants without address information were removed (*n* = 150 in FinnTwin16 and *n* = 53 in FinnTwin12).

### 2.3 Statistical Analysis

To maximize statistical power, two cohorts were combined for the analysis, resulting in a total of 4312 individuals (2353 in FinnTwin16 and 1959 in FinnTwin12), of whom 1211 complete twin pairs (477 MZ and 734 DZ pairs).

### 2.4 Regression Model Between Residential Walkability and BMI

Multiple linear regression was used to examine the association between residential walkability and BMI. Two adjustments were applied: minimally adjusted model, including age (calculated as the difference between response date and date of birth) and sex (from registry data), and fully adjusted model, which additionally included work status (employed including entrepreneur vs. other), education level (postsecondary or lower vs. bachelor/equivalent or above), living status (living with a partner vs. other), and community deprivation *z*‐score (calculated using three social indicators from Statistics Finland [22]). These covariates were identified a priori because of the associations between the living environment and BMI from previous studies [23]. Adjusting for the community deprivation *z*‐score also considers the contextual effect from the community. Modifiable lifestyles were not included as covariates, as they are more likely to act as mediators rather than confounders. Missing covariates were imputed by the median. Standard errors were adjusted using maximum likelihood estimation to account for the twin pair sampling design (cluster effect). To address unobserved genetic and shared familial confounding, a pairwise linear regression model was applied between differences in residential walkability and in BMI between MZ cotwins only. The same adjustments were applied, with covariates calculated as within‐pair differences or discordant. Sex for each pair was still defined as male and female. Age difference was not considered. Robust standard errors were not applied in this model. The R package “lavaan” (version 0.6–19) was used [24].

### 2.5 Univariate Twin Modeling

The classical univariate twin model was applied to the residential walkability to decompose the observed variance into the additive genetic (A), shared environmental (C)/dominant genetic (D), and unique environmental (E) components [25]. The initial model specification was guided by intrapair correlations (ρ) by zygosity; the ADE model was chosen if the ρMZ exceeded twice ρDZ, suggesting no shared environmental influence. There was a path from each component toward the residential walkability, and the square of path coefficients was the unstandardized variance explained by each component. Nested models (AE, CE, E) were subsequently tested by sequentially dropping variance components from the full model. The DE nested models were not considered, since it is biologically implausible. Model fit was evaluated primarily by Akaike information criterion (AIC; smaller is better). The saturated twin model was performed to examine the assumptions of equal means and variances by twin order and zygosity. The residual of residential walkability, regressed by age and sex, was input into the model as the covariate adjusting (residualized). The R package “OpenMx” (version 2.21.13) was used [26].

### 2.6 Bivariate Moderation Twin Modeling

Univariate twin modeling was conducted for BMI to estimate its variance components following the same procedure. Based on these results, a bivariate Cholesky moderation model was applied to assess whether the residential walkability moderated the variances of BMI explained by different components. In addition to standard bivariate Cholesky paths (unique A, C/D, and E paths toward two variables and paths shared by two variables), beta terms were introduced to quantify the extent of moderation on both shared and unique paths toward BMI [27]. The residential walkability and BMI were residualized by age and sex, as covariate adjusting. The R package “OpenMx” (version 2.21.13) was used [26].

## 3 Results

### 3.1 Characteristics of Included Participants

The analytical sample consisted of 4312 participants, and 60% were females (58% in FinnTwin12 and 61% in FinnTwin16) (Table 1). The overall mean age was 43 years (standard deviation [SD]: 5), with FinnTwin12 participants averaging 37 years (SD: 1) and FinnTwin16 participants averaging 47 years (SD: 1). The majority of participants were employed (89%), held a bachelor's degree (or equivalent) or above (62%), and lived with a partner (72%) (Table 1). The overall mean BMI was 26 (SD: 5), being slightly lower in FinnTwin12 (26 [SD: 5]) than in FinnTwin16 (27 [SD: 5]).

**TABLE 1:** Characteristics of participants included in the analysis (*n* = number of individuals).

Characteristics	Combined cohort (individual *n* = 4312)	FinnTwin16 (individual *n* = 2353)	FinnTwin12 (individual *n* = 1959)
Sex, *n* (%)			
Male	1728 (40.1)	912 (38.8)	816 (41.7)
Female	2584 (59.9)	1441 (61.2)	1143 (58.4)
Zygosity, *n* (%)			
Monozygosity	1442 (33.4)	758 (32.2)	684 (34.9)
Dizygosity	2870 (66.6)	1595 (67.8)	1275 (65.1)
Work status, *n* (%)^a^			
Employed (including the entrepreneur)	3841 (89.4)	2114 (91.6)	1696 (86.7)
Other situations	456 (10.6)	196 (8.4)	260 (13.3)
Education level, *n* (%)^b^			
Postsecondary or lower	1623 (37.7)	920 (39.2)	703 (35.9)
Bachelor/equivalent or above	2682 (62.3)	1428 (60.8)	1253 (64.1)
Living status, *n* (%)^c^			
With a spouse or partner	3103 (72.1)	1669 (71.1)	1434 (73.3)
Other situations	1202 (27.9)	679 (28.9)	523 (26.7)
Age, mean (SD)	42.6 (5.2)	47.2 (1.4)	37.2 (1.5)
Body mass index (kg/m^2^), mean (SD)	26.5 (5.1)	27.0 (5.2)	25.9 (4.9)
Residential walkability, mean (SD)	43.4 (12.0)	42.1 (11.2)	45.0 (12.7)

^a^ 12 and 3 participants in FinnTwin16 and FinnTwin12 missed information in the variable of work, respectively.

^b^ 4 and 3 participants in FinnTwin16 and FinnTwin12 missed information in the variable of education, respectively.

^c^ 5 and 2 participants in FinnTwin16 and FinnTwin12 missed information in the variable of living status, respectively.

### 3.2 Association Between Residential Walkability and BMI

**FIGURE 1:**
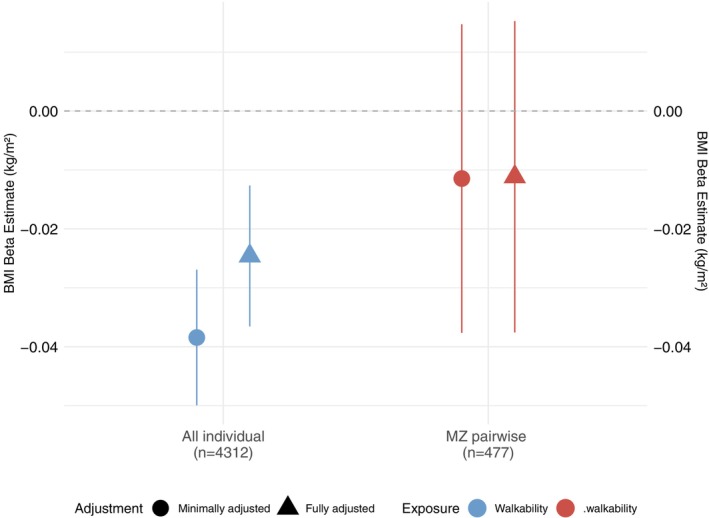
Individual and pairwise association between residential walkability and BMI. The associations between residential walkability and BMI were estimated by multiple linear regression models at both the individual level and within monozygotic (MZ) twin pairs. The minimally adjusted model included age and sex. The fully adjusted model additionally included work status, education level, living status, and community deprivation *z*‐score. Covariates were converted to within‐pair difference or discordance in the MZ pairwise model (age was excluded). [Color figure can be viewed at wileyonlinelibrary.com]

After adjusting for sex and age, among all included individuals, a higher level of residential walkability was significantly associated with lower BMI (coefficient: −0.04, 95% confidence interval [CI]: −0.05, −0.03) (Figure 1). The incremental *R* ^2^ was 0.01 for residential walkability. However, among MZ twin pairs, there was no significant association between the differences in residential walkability and BMI (coefficient: −0.01, 95% CI: −0.04, 0.01). After full adjustment (Figure 1), the results were similar, such that residential walkability was significantly associated with BMI, but the effect size was attenuated (coefficient: −0.02, 95% CI: −0.04, −0.01), among all included individuals. Still, no statistically significant association between the MZ within‐twin‐pair differences in residential walkability and BMI was observed. The examinations of linearity, normality, and heteroscedasticity of minimally adjusted models are presented in Figures S4–S6, respectively.

Sex stratification was conducted as the sensitivity analysis (Table S1). Among male and female individuals, a higher level of residential walkability was significantly associated with lower BMI. However, regardless of sex, pairwise models did not yield any significant results. Another sensitivity analysis stratified the model by the spatial layer of centers and shopping areas in 2019 from the community structure monitoring system of the Finnish Environment Institute to examine potential contextual effects by urbanization [28]. There were 19.3% of participants who lived in centers and shopping areas, so stratification was not performed in the MZ pairwise model. After full adjustment, the association between residential walkability and BMI attenuated to null among participants not living in centers and shopping areas (Table S2).

### 3.3 Genetic Contribution to the Variance of Residential Walkability

The intraclass correlation among MZ pairs (ρMZ) for residential walkability was 0.46 (95% CI: 0.39, 0.53), and ρDZ was 0.34 (95% CI: 0.28, 0.41), indicating selecting the ACE model initially. The saturated model suggested that twin modeling assumptions were met (Table S3). The ACE model was the most optimal for residential walkability, compared to AE, CE, and E nested models (Table 2). The additive genetic component (A) accounted for 22% (95% CI: 0.04, 0.40) of the variation in residential walkability. The shared environmental component (C) accounted for 22% (95% CI: 0.08, 0.36) of the variation. The unique environmental component (E) accounted for the largest proportion of the variation, 56% (95% CI: 0.51, 0.61).

**TABLE 2:** Standardized variance components for residential walkability estimated by univariate twin modeling^a^.

Model	Standardized variance (95% CI)			AIC	*p* ^b^
	A (additive genetic)	C (common environmental)	E (unique environmental)		
ACE	0.22 (0.04, 0.40)	0.22 (0.08, 0.36)	0.56 (0.51, 0.61)	18664.05	Ref.
AE	0.48 (0.42, 0.53)	/	0.52 (0.47, 0.58)	18671.15	< 0.01
CE	/	0.37 (0.32, 0.42)	0.63 (0.58, 0.68)	18667.51	0.02
E	/	/	1	18846.73	< 0.01

^a^ The total variance of residential walkability was decomposed into additive genetic (A), shared environmental (C), and unique environmental (E) components using univariate twin modeling. Values represent standardized proportions of total variance.

^b^ The *p* value was for the likelihood test between models.

### 3.4 Moderation by Residential Walkability on Genetic and Environmental Contributions to BMI

**FIGURE 2:**
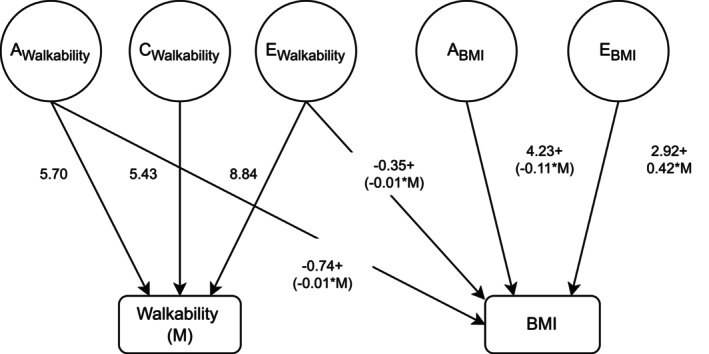
Diagram of the bivariate moderation twin model examining residential walkability and BMI with model output. The bivariate moderation twin model was used to examine the genetic and environmental influences underlying residential walkability and BMI and their potential moderation effects. Observed variables are depicted as rectangles, and latent variables representing additive genetic (A) and unique environmental (E) components are shown as circles. Single‐headed arrows indicate directional effects of latent components on observed traits, whereas curved double‐headed arrows represent correlations between latent factors. The model allows for the moderation of genetic and environmental components as a function of the moderator variable. Numerical values displayed along the paths represent the estimated path coefficients from the final fitted bivariate moderation twin model. The residential walkability and BMI were residualized by age and sex, as covariate adjusting.

**FIGURE 3:**
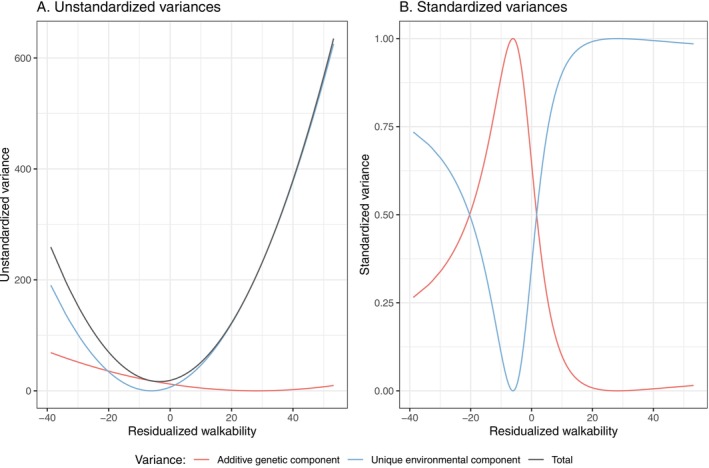
Genetic and environmental components of BMI from the bivariate moderation twin model. The additive genetic (A) and unique environmental (E) components of BMI across the moderator (residential walkability) level were estimated by the bivariate moderation twin model. Panel A shows the unstandardized variance components, and Panel B shows the corresponding standardized variance proportions. Residential walkability and BMI were residualized for age and sex, as covariate adjusting. [Color figure can be viewed at wileyonlinelibrary.com]

The AE model was identified as the best‐fitting model for BMI (Table S4), and its twin modeling assumptions were tested (Table S3). The initial bivariate moderation model combined an ACE model for residential walkability and an AE model for BMI, including shared A, C, and E paths. Since BMI showed only A and E components, the shared C path was constrained as another nested model. The nested model was better in fit (AIC difference > 2). Figure 2 presents the estimated path coefficients and beta terms of the nested model as the final result. The beta value on the unique A path toward BMI was slightly smaller than the beta value on the corresponding E path. Figure 3 displays projected unstandardized and standardized A and E components along with residential walkability, calculated based on all values of residential walkability (regardless of twin order), based on the structural equation model in Figure 2. With increasing residential walkability, the total variance varied with the contribution of E, and the contribution of A flatly decreased. Two intersections between A and E lines indicate G×E interaction. When individuals lived in areas with an average or below average level of residential walkability (residualized residential walkability ranged between −20 and 1.5), the influence of A was much stronger, while if they lived in a more extreme area (either much higher or lower residential walkability than average), the E contributed more (almost 100%).

## 4 Discussion

In a sample of over 4000 middle‐aged adults from two Finnish twin cohorts, this study examined the interplay between neighborhood walkability, genetics, and BMI. Higher residential walkability was associated with lower BMI. However, this relationship was not independent of genetic and shared early‐life environmental influences. The additive genetic component explained ~22% of the variation in residential walkability when participants were in their mid‐30s or 40s. Notable G×E interaction was observed, wherein genetic contributions to BMI were more pronounced among individuals living in areas with average or a bit lower than average levels of residential walkability, while the influence of the unique environmental component was greater among those living in areas characterized by either very low or very high walkability.

Many studies supported the protective effect of high residential walkability against unhealthy weight outcomes. The aforementioned 2019 systematic review generally supported this association [10], and some studies, not included in the review, also reported similar findings. For example, the North West Adelaide Health Study in Australia (3205 participants) found that higher walkability was associated with reduced abdominal obesity [29], and there was a negative association between walkability and overweight or obesity among 4303 Latino adults in the United States [30]. Nonlinear relationships have also been observed. Based on the 2006 Nurses' Health Study in the Northeastern United States with over 23,000 elderly females, BMI was found to increase at lower levels of walkability but decrease at higher levels [31]. Another US study with 4870 individuals reported BMI increases with walkability up to a certain threshold [32]. The threshold or nonlinear effect may be due to the contextual effect of the level of urbanization, as shown in the sensitivity analysis. Despite these findings, evidence remains inconsistent. The 2019 systematic review noted that 3 of 10 included studies found no significant association [10]. A cross‐sectional study of adults aged 65–97 years in the United States and a meta‐analysis focusing on children found no association between walkability and weight outcomes, although in populations of different age groups [33, 34]. These inconsistencies may partly reflect that highly walkable neighborhoods often have a higher density of unhealthy food retail, which suggests that diet could be a possible mechanism [35]. Together with the null results observed in our pairwise analyses, this suggests that genetic factors may also influence the relationship between walkability and BMI.

The 22% of the variation in residential walkability, in participants in their mid‐30s or mid‐40s, was explained by the additive genetic component and aligned with estimates in WSTR (mean age: 37) (19%) [13]. This may further suggest the presence of a nontargeted G×E correlation, wherein individuals' genetic predispositions influence their likelihood of selecting to reside in more or less walkable neighborhoods. Therefore, the quantitative genetic study designs, such as Mendelian randomization, shall be more cautiously employed in the field of environmental health. Heritable traits (e.g., socioeconomic status [36], cognitive ability [37]) can influence residential selection, violating the assumption of no unmeasured confounding by determinants of residential location. In addition, some single‐nucleotide polymorphisms may also directly influence BMI‐related traits, raising concerns of pleiotropy.

The G × E effect differed between our findings and those from WSTR. We identified a pattern suggesting that within a certain range of walkability, genetic influences on BMI were much stronger. In contrast, WSTR found that higher walkability attenuated genetic risk for high BMI in a more linear fashion [13]. An analysis of 9918 women from the Utah Population Database demonstrated that familial risk, measured by siblings' BMI, enhanced the association between low walkability and increased obesity risk [38]. Other research has broadened the scope to obesogenic environments. In the UK Biobank, individuals with a higher genetic risk of obesity, measured by polygenic risk scores or individual variants, were more strongly affected by proximity to fast‐food outlets [39]. A twin study in England and Wales showed that BMI heritability at age 4 for those living in higher‐risk obesogenic home environments was more than twice that for those living in lower‐risk obesogenic home environments [40]. One possible explanation for the observed G × E effect is that MZ twins tend to have closer relationships and therefore often live physically closer to each other. This pattern applies to a lesser extent to DZ twins, explaining the shared environmental component. In our data, the average distance between cotwins' geocodes, used to merge residential walkability, was smaller in MZ (80.6 km [SD: 134.5]) than in DZ (105.8 km [SD: 148.8]) pairs, although a *t*‐test indicated this difference was not statistically significant.

The implication of G×E interplay extends beyond recognizing that genetic influence alone, and it also emphasizes that walkability, as a modifiable environmental factor, has the capacity to be improved to overcome genetic susceptibility and promote health on a population scale through substantial investment. Natural experiments of college students in the United States randomly assigned to dormitories with varying obesogenic characteristics (e.g., distance to food outlets or fitness facilities) have shown that more favorable environments are associated with reduced weight gain among females. The natural experiment design reduced the genetic confounding to some extent, as well [41]. Policy makers are encouraged to integrate walking‐friendly design into urban planning as an effective strategy for obesity prevention [42].

There were some limitations. First, the relatively small number of MZ pairs limited the statistical power of within‐pair analyses, partly due to the loss of follow‐up of one cotwin in the pair. Collaboration within the global twin research community could help increase sample sizes and boost robust findings. The second limitation is the reverse causality, meaning that earlier BMI may influence residential choices and, consequently, exposure to walkable environments. Repeated measures on both BMI and walkability can clarify the directionality of this relationship. Further longitudinal investigation is warranted. Third, the generalizability of our findings is limited to Finland, a country with the highest walkability in Europe and relatively modest urban–rural differences. As the walkability indicator will be publicly available, we encourage replication of this study across Europe. Fourth, the weight and height for BMI were inquired via self‐reporting, which may be subject to recall bias and measurement error. In addition, BMI, while widely used in epidemiological studies, does not fully capture fat distribution or body composition and may not perfectly reflect obesity status. Further validation that incorporates objectively measured anthropometric data with a composite index by trained health professionals is needed. Fifth, lifestyles (not included as covariates) are partly heritable, and the null findings from MZ pairwise models might imply that early shared genetic factors, confounding the association between residential walkability and BMI, may influence lifestyle as well [43]. This highlights the complexity between the environment, genetics, behavior, and health, encouraging more comprehensive and integrative approaches in the future.

## 5 Conclusion

This study highlights the complex interplay between residential walkability, genetics, and BMI in Finland. Higher walkability was associated with lower BMI, but this relationship seems to be influenced by both genetic predispositions and early shared environmental factors when people are middle‐aged. The genetic component of walkability, as the gene–environment correlation, should be considered in future genetic studies. Coupled with evidence of gene–environment interaction, public health interventions shall account for both genetic susceptibility and modifiable environmental factors. Since genetic predisposition is hard to address, improving the living environment, such as the element of walkability, remains a key strategy for reducing obesity risk.

## Author Contributions

**Zhiyang Wang:** conceptualization, methodology, formal analysis, writing – original draft. **Bram Berntzen:** conceptualization, methodology, formal analysis, writing – review and editing. **Nishit Patel:** data curation, writing – review and editing. **Stephanie Zellers:** formal analysis, writing – review and editing. **Sari Aaltonen:** data curation, writing – review and editing. **Danielle Dick:** data curation, writing – review and editing, funding acquisition. **Karri Silventoinen:** data curation, writing – review and editing. **Jeroen Lakerveld:** conceptualization, data curation, supervision; writing – review and editing. **Jaakko Kaprio:** conceptualization, data curation, supervision, writing – review and editing, funding acquisition.

## Funding

Data collection in FinnTwin12 has been supported by the National Institute on Alcohol Abuse and Alcoholism (grants AA‐12502, AA‐00145, and AA‐09203 to Richard J. Rose and AA015416 to Danielle Dick and Jessica Salvatore) and the Academy of Finland (grants 100499, 205585, 118555, 141054, 264146, 308248, 312073, 336823, and 352792 to Jaakko Kaprio). Jaakko Kaprio acknowledges support by the Academy of Finland (grants 265240 and 263278). The latest data collection in FinnTwin16 was supported by the Academy of Finland (grants 336823 and 352792 to Jaakko Kaprio). This project, in particular the developed walkability index, is a part of the OBCT project (www.obct.nl), which has received funding from the European Union's Horizon Europe research and innovation program under grant agreement No. (101080250).

## Conflicts of Interest

The authors declare no conflicts of interest.

## Supporting information


**Table S1:** Sex‐stratified associations between residential walkability and BMI by multiple linear regression models at both the individual level and within monozygotic (MZ) twin pairs. The minimal adjustment included age and sex. The full adjustment also included work status, education level, living status, and community deprivation *z*‐score. Covariates were converted to within‐pair difference or discordance in the MZ pairwise model (age was excluded).
**Table S2:** Stratified individual‐level associations between residential walkability and BMI by the level of urbanization. The minimal adjustment included age and sex. The full adjustment also included work status, education level, living status, and community deprivation *z*‐score.
**Table S3:** Saturated models for assumption testing of univariate twin modeling through constrain expected means or (and) variances across twins.
**Table S4:** Standardized variance components for BMI by univariate twin modeling.
**Figure S1:** Scatter plots for linearity test for the minimally adjusted regression model between residential walkability level and BMI among all individuals.
**Figure S2:** Scatter plots for linearity test for the minimally adjusted regression model between residential walkability level and BMI among MZ pairs.
**Figure S3:** Quantile‐quantile plots for normality test for the minimally adjusted regression model between residential walkability level and BMI among all individuals.
**Figure S4:** Quantile‐quantile plots for normality test for the minimally adjusted regression model between residential walkability level and BMI among MZ pairs.
**Figure S5:** Residual plots for heteroscedasticity test for the minimally adjusted regression model between residential walkability level and BMI among all individuals.
**Figure S6:** Residual plots for heteroscedasticity test for the minimally adjusted regression model between residential walkability level and BMI among MZ pairs.

## Data Availability

The FinnTwin12 and FinnTwin16 data are not publicly available due to the restrictions of informed consent. However, the data are available through the Institute for Molecular Medicine Finland (FIMM) Data Access Committee (DAC) (fimm-dac@helsinki.fi) for authorized researchers who have institutional review board/ethics approval and an institutionally approved study plan. To ensure the protection of privacy and compliance with national data protection legislation, a data use/transfer agreement is needed, the content and specific clauses of which will depend on the nature of the requested data. Requests will be addressed in a reasonable time frame (generally 2 to 3 weeks), and the primary mode of data access is by either personal visit or remote access to a secure server. High‐resolution walkability data for Finland (as used in the current study) or other European countries can be obtained via the OBCT coordinating office (www.obct.nl).
