# Supplementary material for: Gene–Environment Interplay Between Residential Walkability and BMI: A Finnish Twin Study
Source: Obesity (Silver Spring). 2026 Apr 27;34(5):1163–72. doi: 10.1002/oby.70191 (PMC13116020; doi:10.1002/oby.70191)

Table S1: Sex-stratified associations between residential walkability and BMI by multiple linear regression models at both the individual level and within monozygotic (MZ) twin pairs. The minimal adjustment included age and sex. The full adjustment also included work status, education level, living status, and community deprivation z-score. Covariates were converted to within-pair difference or discordance in the MZ pairwise model (age was excluded).

| Model | Coefficient (95% CI) | |
| --- | --- | --- |
|  | Minimally adjusted model | Fully adjusted model |
| *Male* | | |
| All individual | -0.03 (-0.05, -0.02)* | -0.04 (-0.06, -0.03)* |
| MZ pairwise | >-0.01 (-0.04, 0.04) | -0.02 (-0.05, 0.02) |
| *Female* | | |
| All individual | -0.02 (-0.04, -0.01)* | -0.03 (-0.04, -0.01)* |
| MZ pairwise | <0.01 (-0.04, 0.04) | -0.02 (-0.05, 0.02) |

* P-value < 0.05

Table S2: Stratified individual-level associations between residential walkability and BMI by the level of urbanization. The minimal adjustment included age and sex. The full adjustment also included work status, education level, living status, and community deprivation z-score.

| Model among all individuals | Coefficient (95% CI) | |
| --- | --- | --- |
|  | Minimally adjusted model | Fully adjusted model |
| Living in center and shopping areas (N. = 830) | -0.05 (-0.08, -0.03)* | -0.03 (-0.06, -0.01)* |
| Not living in center and shopping areas (N. = 3482) | -0.;02 (-0.04, -0.03)* | -0.01 (-0.03, 0.01) |

* P-value < 0.05

Table S3: Saturated models for assumption testing of univariate twin modeling through constrain expected means or (and) variances across twins.

| **Variable** | **Model** | **Estimated parameters** | **-2 loglikelihood  (-2ll)** | **Degree of freedom (df)** | **AIC** | **Difference of -2ll** | **Difference of df** | **Two-sided P-value ^a^** |
| --- | --- | --- | --- | --- | --- | --- | --- | --- |
| Residential walkability level | Saturated | 25 | 18649.99 | 2397 | 18699.99 | Ref. | | |
|  | Constrain expected Means to be equal across twin order | 21 | 18654.94 | 2401 | 18696.94 | 4.95 | 4 | 0.29 |
|  | Constrain expected Means and Variances to be equal across twin order | 17 | 18668.08 | 2405 | 18702.08 | 18.10 | 8 | 0.02 |
|  | Constrain expected Means and Variances to be equal across twin order and zygosity | 13 | 18675.48 | 2409 | 18701.48 | 25.49 | 12 | 0.01 |
| BMI | Saturated | 25 | 14212.73 | 2397 | 14262.73 | Ref. | | |
|  | Constrain expected Means to be equal across twin order | 21 | 14216.16 | 2401 | 14258.16 | 3.43 | 4 | 0.49 |
|  | Constrain expected Means and Variances to be equal across twin order | 17 | 14220.94 | 2405 | 14254.94 | 8.21 | 8 | 0.41 |
|  | Constrain expected Means and Variances to be equal across twin order and zygosity | 13 | 14226.46 | 2409 | 14252.46 | 13.73 | 12 | 0.32 |

^a^ The p-value was for the likelihood test between models.

Table S4: Standardized variance components for BMI by univariate twin modelling^a^

| **Model** | **Standardized variance (95% confidence interval)** | | | **AIC** | **P-value ^b^** |
| --- | --- | --- | --- | --- | --- |
|  | A (additive  genetic) | D (dominance genetic) | E (unique environmental) |  |  |
| ADE | 0.67 (0.41, 0.91) | 0.08 (-0.17, 0.34) | 0.26 (0.22, 0.30) | 14282.45 | Ref. |
| AE | 0.74 (0.70, 0.77) | / | 0.26 (0.23, 0.30) | 14280.81 | 0.55 |
| E | / | / | 1 | 14756.06 | <0.01 |

^a^ The total variance of residential walkability was decomposed into additive genetic (A), shared environmental (C), and unique environmental (E) components using univariate twin modeling. Values represent standardized proportions of total variance.

^b^ The p-value was for the likelihood test between models.

Figure S1: Scatter plots for linearity test for the minimally adjusted regression model between residential walkability level and BMI among all individuals


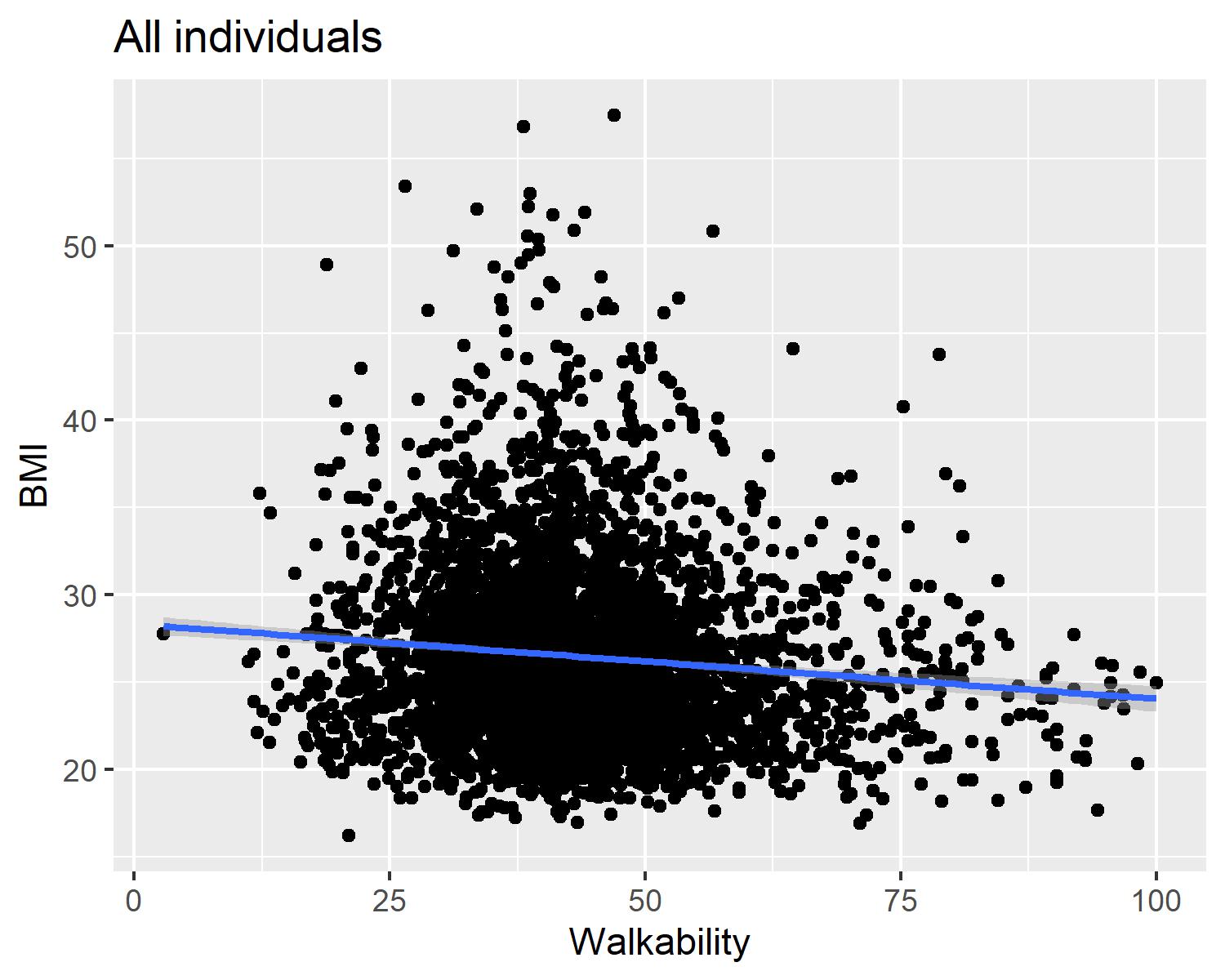


Figure S2: Scatter plots for linearity test for the minimally adjusted regression model between residential walkability level and BMI among MZ pairs


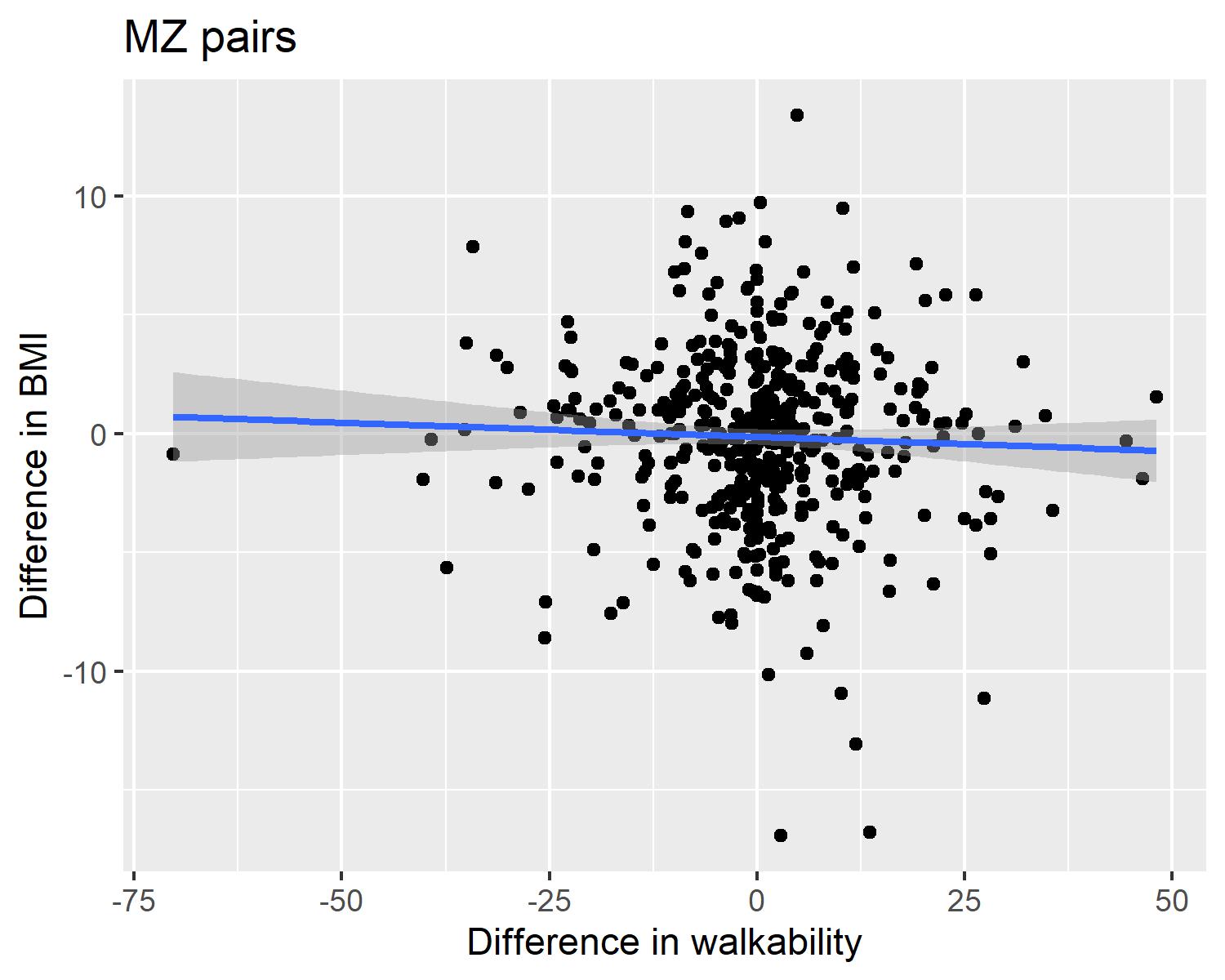


Figure S3: Quantile-quantile plots for normality test for the minimally adjusted regression model between residential walkability level and BMI among all individuals


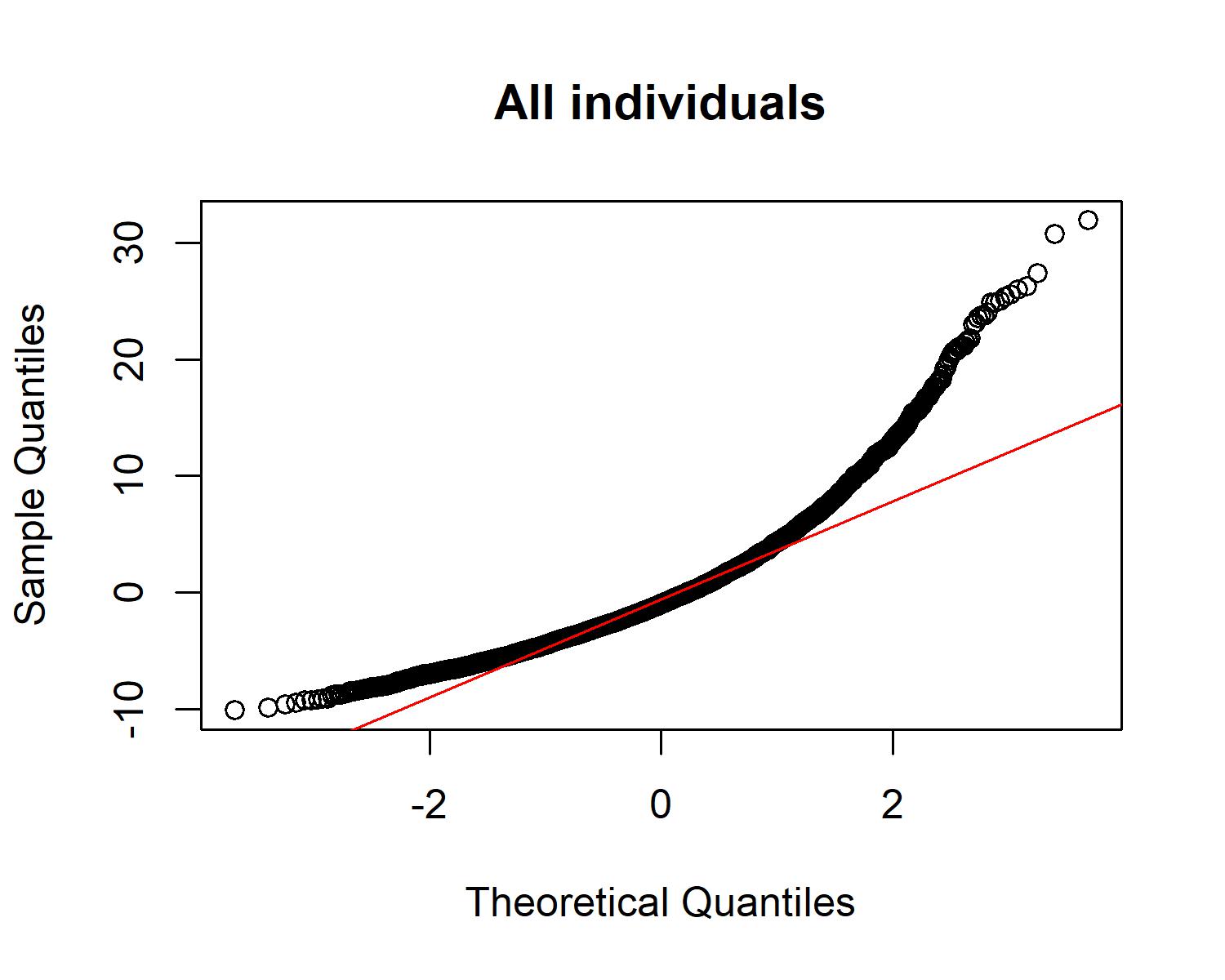


Figure S4: Quantile-quantile plots for normality test for the minimally adjusted regression model between residential walkability level and BMI among MZ pairs


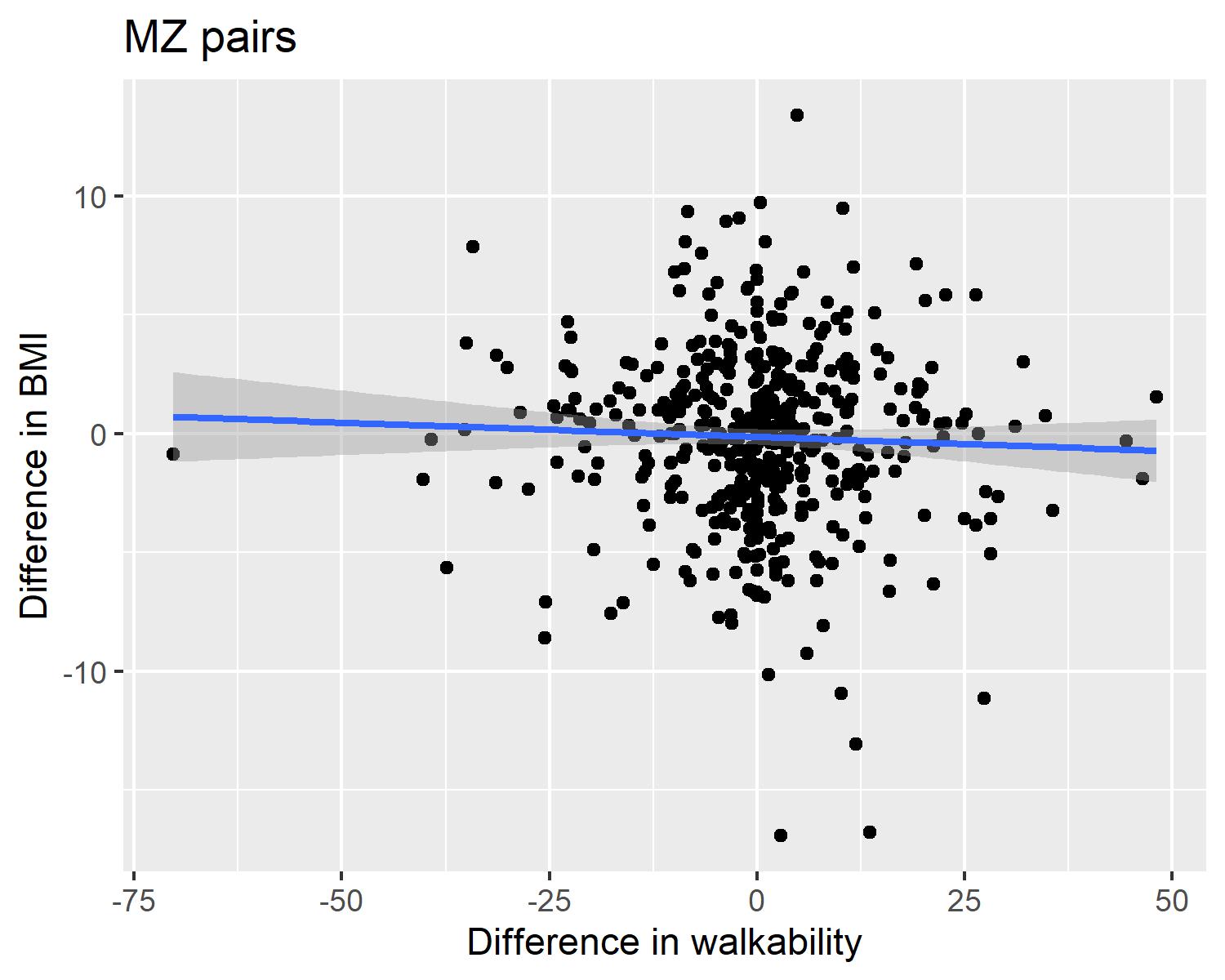


Figure S5: Residual plots for heteroscedasticity test for the minimally adjusted regression model between residential walkability level and BMI among all individuals


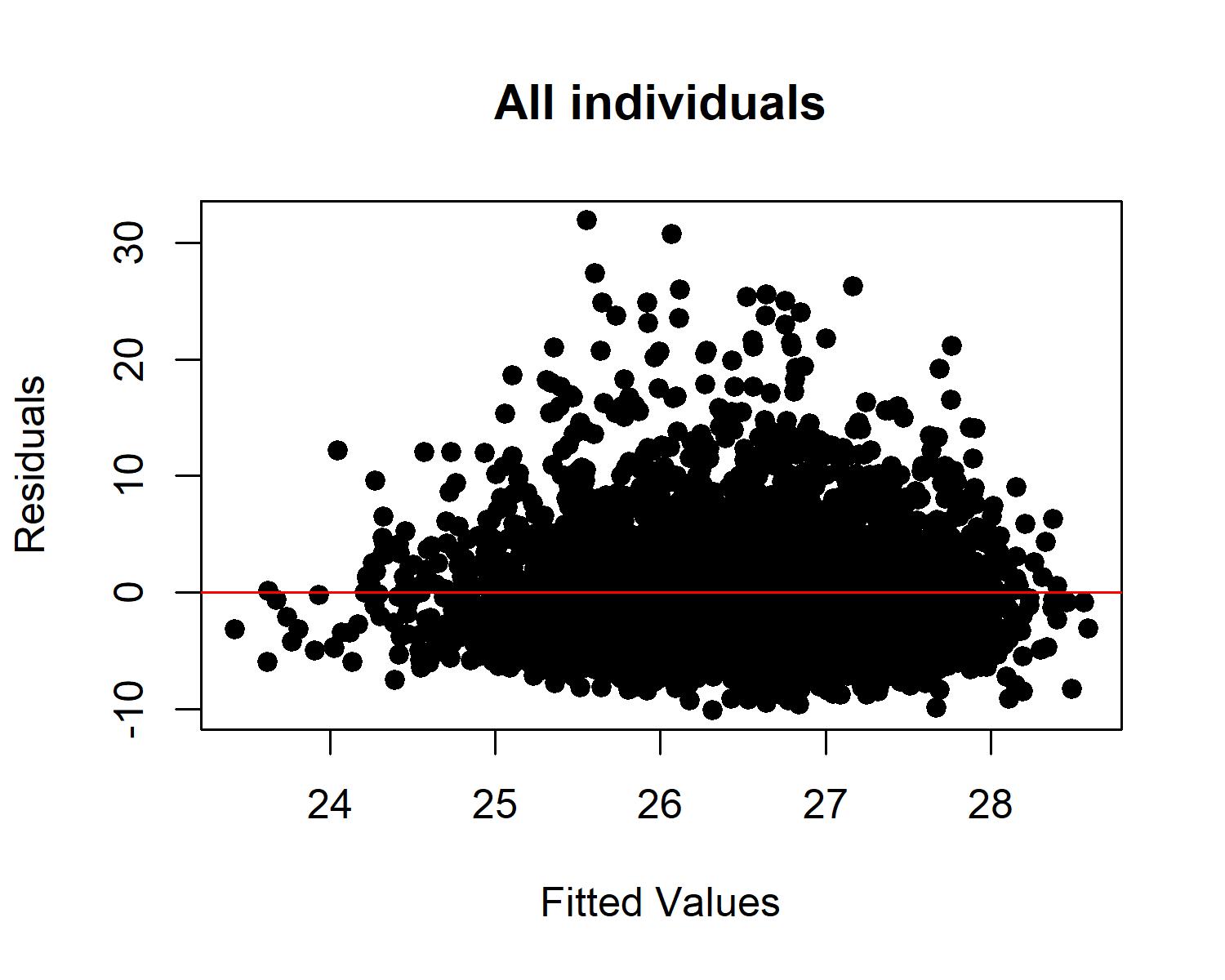


Figure S6: Residual plots for heteroscedasticity test for the minimally adjusted regression model between residential walkability level and BMI among MZ pairs


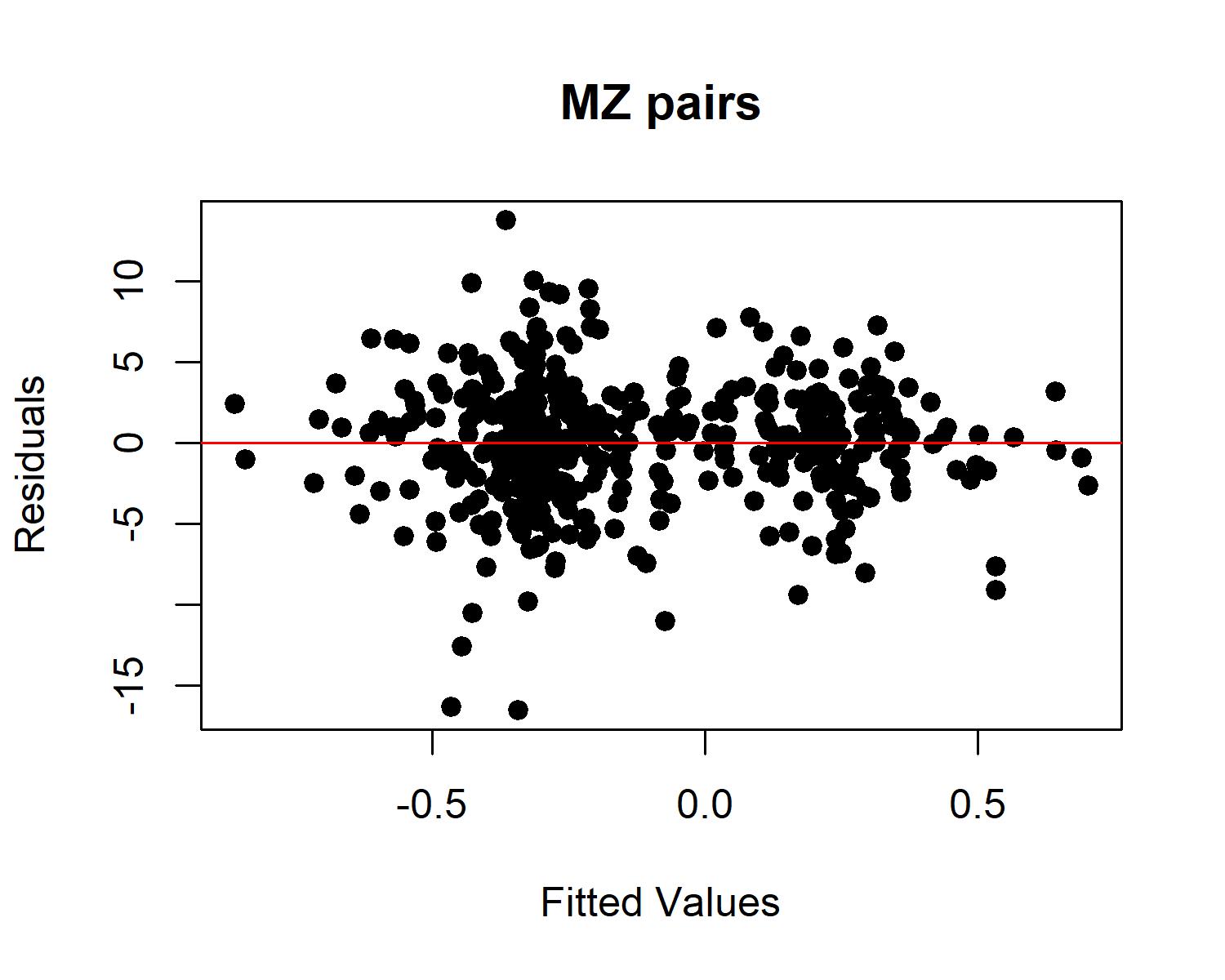

Supplement: Supplementary file 1 — Table S1: Sex‐stratified associations between residential walkability and BMI by multiple linear regression models at both the individual level and within monozygotic (MZ) twin pairs. The minimal adjustment included age and sex. The full adjustment also included work status, education level, living status, and community deprivation z‐score. Covariates were converted to within‐pair difference or discordance in the MZ pairwise model (age was excluded). Table S2: Stratified individual‐level associations between residential walkability and BMI by the level of urbanization. The minimal adjustment included age and sex. The full adjustment also included work status, education level, living status, and community deprivation z‐score. Table S3: Saturated models for assumption testing of univariate twin modeling through constrain expected means or (and) variances across twins. Table S4: Standardized variance components for BMI by univariate twin modeling. Figure S1: Scatter plots for linearity test for the minimally adjusted regression model between residential walkability level and BMI among all individuals. Figure S2: Scatter plots for linearity test for the minimally adjusted regression model between residential walkability level and BMI among MZ pairs. Figure S3: Quantile‐quantile plots for normality test for the minimally adjusted regression model between residential walkability level and BMI among all individuals. Figure S4: Quantile‐quantile plots for normality test for the minimally adjusted regression model between residential walkability level and BMI among MZ pairs. Figure S5: Residual plots for heteroscedasticity test for the minimally adjusted regression model between residential walkability level and BMI among all individuals. Figure S6: Residual plots for heteroscedasticity test for the minimally adjusted regression model between residential walkability level and BMI among MZ pairs. [file OBY-34-1163-s001.docx]
